# Supplementary material for: Content-rich biological network constructed by mining PubMed abstracts
Source: BMC Bioinformatics. 2004 Oct 8;5:147. doi: 10.1186/1471-2105-5-147 (PMC528731; doi:10.1186/1471-2105-5-147)
Supplement: Additional File 2 — The original results of the above study (non-essential files are deleted to keep the file size under the limit set by BMC bioinformatics). [file 1471-2105-5-147-S2.bz2 › chilibotAdditionalFile2/dip40/428ID7622448E10358/html/CCR1_CCL3.html]

 


 **CCR1** and **CCL3** 
  
Found 74 abstracts in PubMed, retrieved 40.  
 

 What does Google say? 
 PDF only 
| .edu only 

---

**Interactive relationship** (e.g. stimulation, inhibition, etc)

**Stimulatory relationship**- Eosinophils from these donors showed increased eosinophil  **CCR1**  expression and also underwent  **CCL3**  mediated chemotaxis and up regulation of CD11b.  Ref: 12794150 J Immunol, 2003
- Thus, IL 1 may enhance the local production of  **CCL3** , which may interact with  **CCR1**  expressed on hepatoma cells, in an autocrine and or paracrine manner.  Ref: 12651617 Am J Pathol, 2003
- CCL9, which acts through  **CCR1** , stimulated cytoplasmic motility and polarization in osteoclasts, identical to that previously observed in response to  **CCL3**  MIP 1alpha, which also acts through  **CCR1**  and is chemotactic for osteoclasts.  Ref: 12397598 J Cell Biochem, 2002
- Survival of CCR5 mice was equivalent to that of controls, indicating that the enhanced susceptibility of  **CCL3**  mice to K. pneumoniae is mediated via another  **CCL3**  receptor, presumably  **CCR1** .  Ref: 11553580 Infect Immun, 2001
- Increase of  **CCR1**  and CCR5 expression and enhanced functional response to MIP 1 alpha  [ **CCL3** ]  during differentiation of human monocytes to macrophages.  Ref: 11272275 J Leukoc Biol, 2001
- Moreover,  **CCL3**  L1, but not  **CCL3** , is susceptible to cleavage by CD26, creating a truncated 2 form with enhanced affinity for  **CCR1**  and CCR5.  Ref: 12355456 Eur J Immunol, 2002
**Neutral relationship**- However, the remaining expression of  **CCR1**  and CCR5 correlated reciprocally with an ongoing unresponsiveness to the  **CCR1**  and CCR5 agonists  **CCL3**  macrophage inflammatory protein 1alpha MIP 1alpha, CCL4 MIP 1beta, and CCL5 regulated on activation, normal T expressed and secreted RANTES, all chemokines binding to these two receptors.  Ref: 12885942 J Leukoc Biol, 2003
- One such receptor is  **CCR1**  which is a receptor for CC chemokines, such as CCL5 RANTES and  **CCL3**  MIP 1alpha.  Ref: 12769747 Curr Pharm Des, 2003
- Potential Interaction between  **CCR1**  and Its Ligand,  **CCL3** , Induced by Endogenously Produced Interleukin 1 in Human Hepatomas.  Ref: 12651617 Am J Pathol, 2003

**Non-interactive relationship** (e.g. studied together, co-existance, homology, etc.)

- For examples, Langerhans cells migrate selectively to MIP 3 alpha via CCR6, blood CD11c DC to MCP chemokines via CCR2, monocytes derived DC respond to MIP 1 alpha  [ **CCL3** ]  beta via  **CCR1**  and CCR5, while blood CD11c DC precursors do not respond to any of these chemokines.  Ref: 11155441 Springer Semin Immunopathol, 2000
- We also evaluated the expression of the  **CCR1**  ligand macrophage inflammatory protein 1alpha MIP 1alpha  **CCL3** .  Ref: 12541321 J Comp Neurol, 2003
- Moreover, the forskolin mediated increases in intracellular cAMP concentrations were inhibited by the ligands for  **CCR1** ,  **CCL3** , CCL4, and CCL5, suggesting that the expressed  **CCR1**  was functional.  Ref: 12651617 Am J Pathol, 2003
- To test the in vivo relevance of this, we used a murine melanoma system and knockout mice to investigate the function of the chemokine receptor CCR5 and its ligands, CCR  [ **CCR1** ]  ligand CCL 3  [ **CCL3** ]  and CCL5.  Ref: 12682253 J Immunol, 2003
- These results suggest that the MIP 1 alpha  [ **CCL3** ]   **CCR1**  mediated acute inflammatory response protects mice by delaying the lethal sequelae of infection.  Ref: 10946298 J Immunol, 2000
- The most active chemokines for resting Th IL 10, in terms of calcium mobilization and in vitro migration, were in order of potency CCL2 monocyte chemoattractant protein 1, CCR2 ligand, CCL4 macrophage inflammatory protein 1beta, CCR5 ligand,  **CCL3**  macrophage inflammatory protein 1alpha,  **CCR1**  5 ligand, CCL17 thymus and activation regulated chemokine, CCR4 ligand, CCL1 I 309, CCR8 ligand, CXCL12 stromal derived factor 1, CXCR4, and CCL11 eotaxin, CCR3 ligand.  Ref: 11145678 J Immunol, 2001
- An abortive ligand induced activation of  **CCR1**  mediated downstream signaling event and a deficiency of CCR5 expression are associated with the hyporesponsiveness of human naive CD4 T cells to  **CCL3**  and CCL5.  Ref: 12055240 J Immunol, 2002
- The expression of CXCR4 and  **CCR1**  and the migration to their ligands, SDF 1, and RANTES and MIP 1 alpha  [ **CCL3** ] , respectively, were also demonstrated in primary MM cells.  Ref: 12529679 Leukemia, 2003
- In contrast,  **CCL3** , a ligand of  **CCR1**  and CCR5, induced rapid uptake of FITC dextran by immature DCs but not mature DCs.  Ref: 12609829 Blood< MedlineTA>Blood, 2003
- Our studies showed that LD infection caused the upregulation of three beta chemokines macrophage inflammatory protein 1 alpha  [ **CCL3** ]  MIP 1alpha, MIP 1beta and RANTES regulated on activation normal T cell expressed and secreted, one alpha chemokine interleukin 8 IL 8 and the CC chemokine receptor 5 CCR5 but not  **CCR1** , as evident from reverse transcriptase polymerase chain reaction RT PCR analysis.  Ref: 12662300 Scand J Immunol, 2003
- Here we examine the role of MIP 1 alpha  [ **CCL3** ]  in the pathogenesis of this disease using mice deficient in MIP 1 alpha  [ **CCL3** ]  or its receptor,  **CCR1** .  Ref: 10946298 J Immunol, 2000
- In EAE protected mice, 17 beta estradiol strongly inhibited mRNA expression of the chemokines RANTES, MIP 1 alpha  [ **CCL3** ] , MIP 2, IP 10, and MCP 1, and of the chemokine receptors  **CCR1** , CCR2 and CCR5 at both time points.  Ref: 11550221 J Neurosci Res, 2001
- The chemokine macrophage inflammatory protein 1 alpha  [ **CCL3** ]  and its receptor  **CCR1**  control pulmonary inflammation and antiviral host defense in paramyxovirus infection.  Ref: 10946298 J Immunol, 2000
- In vivo neutralization of the  **CCR1**  ligand, macrophage inflammatory protein 1alpha  **CCL3** , resulted in less encephalitogenic CD4 T cell CNS infiltration.  Ref: 11746391 J Neurosci Res, 2001
- In parallel,  **CCR1**  and CCR5 expression is down regulated and CXCL8, CCL2,  **CCL3**  and CCL5 production is up regulated.  Ref: 12548563 Eur J Immunol, 2003
- After 2 to 4 weeks, L. amazonensis infected mice had significantly delayed and depressed expression of inflammatory cytokines interleukin 12 IL 12, gamma interferon, IL 1alpha, IL 1beta, CC chemokines CC chemokine ligand 3  [ **CCL3** ]   **CCL3**  macrophage inflammatory protein 1alpha MIP 1alpha, CCL4 MIP 1beta, CCL5 RANTES, MIP 2, and chemokine receptors  **CCR1** , CCR2, CCR5 in foot tissues and draining lymph nodes compared to the expression in L. major infected controls.  Ref: 12874303 Infect Immun, 2003
- Although only a minority of the cell lines responded by calcium mobilization after agonist stimulation, a migratory response to the  **CCR1**  ligands RANTES and MIP 1 alpha  [ **CCL3** ]  was obtained in 5 6 and 4 6, respectively, of the cell lines tested.  Ref: 12529679 Leukemia, 2003
- A study on the density and distribution of uterine Natural Killer cells at mid pregnancy in mice genetically ablated for CCR2, CCR  [ **CCR1** ]  5 and the CCR5 receptor ligand, MIP 1 alpha  [ **CCL3** ] .  Ref: 11137111 J Reprod Immunol, 2001
- Thus, a failure of the ligand induced activation of  **CCR1**  mediated downstream signaling event as well as a deficiency of CCR5 expression may be involved in the hyporesponsiveness of naive CD4 T cells to  **CCL3**  and CCL5.  Ref: 12055240 J Immunol, 2002
